# Barium-Strontium Titanate/Porous Glass Structures for Microwave Applications

**DOI:** 10.3390/ma13245639

**Published:** 2020-12-10

**Authors:** Andrey Tumarkin, Natalya Tyurnina, Zoya Tyurnina, Nikolay Mukhin, Olga Sinelshchikova, Alexander Gagarin, Sergey Sviridov, Andrey Drozdovsky, Eugeny Sapego, Ivan Mylnikov

**Affiliations:** 1 Department of Physical Electronics and Technology, Electrotechnical University, 5 prof. Popov str., 197376 St. Petersburg, Russia; aggagarin@etu.ru (A.G.); drozdovskiyav@gmail.com (A.D.); eugenysapego@yandex.ru (E.S.); mylnikov.il@gmail.com (I.M.); 2 Institute of Silicate Chemistry, Adm. Makarova emb., 2, 199155 St. Petersburg, Russia; turnina.ng@iscras.ru (N.T.); turnina.zg@iscras.ru (Z.T.); sinelshikova@mail.ru (O.S.); sviridov@iscras.ru (S.S.); 3 Institute for Micro and Sensor Systems, Otto-von-Guericke-University Magdeburg, Universitätspl. 2, 39106 Magdeburg, Germany; mukhin.nikolay.v@gmail.com; 4 Department of Engineering, University of Applied Sciences Brandenburg, Magdeburger Str. 50, 14770 Brandenburg an der Havel, Germany

**Keywords:** glass-ceramic structures, barium-strontium titanate, filling of porous material, microwave

## Abstract

Based on porous silicate glasses obtained by ion exchange, glass-ceramic materials containing a solid solution of barium-strontium titanate with a dielectric constant of more than 100 at microwaves, were synthesized for the first time. Glass-ceramic structures were studied using X-ray diffraction, secondary electron microscopy, Mössbauer spectroscopy and porometry methods. Electrical characteristics such as permittivity and losses of as-prepared and annealed in oxygen medium samples were also investigated at microwaves. It was shown that the method of obtaining porous glasses, due to ion exchange between KFeSi glass and LiNO_3_ and NaNO_3_ melts, allows for controlling a wide range of pore sizes and makes it possible to form glass porous structures with pores of the required size. The efficiency of the process of filling a porous matrix with a ferroelectric filler was investigated and the average depth of its penetration was estimated. It was shown that annealing glass-ceramic structures in an oxygen environment had a positive effect on their structural and electrical characteristics. Glass-ceramic structures demonstrate a significant increase in permittivity and a decrease in losses after high-temperature treatment in oxygen.

## 1. Introduction

Ferroelectric (FE) materials are of great interest for microwave electronics because of their nonlinear response to an electric field. On the basis of the ferroelectric materials, microwave devices such as tunable capacitors, delay lines, phase shifters, etc. [1,2,3] have been actively developed.

However, like any functional materials, ferroelectrics have some disadvantages that limit their use in microwave devices. The weak points of FE materials are rather high microwave losses, a strong dependence of properties on temperature, and difficulties in matching a material with a large dielectric permittivity with microwave circuits [4,5].

One of the ways to minimize the above-mentioned disadvantages and improve the functional characteristics of FE materials is to create composite structures that combine ferroelectrics and linear dielectrics [6,7,8,9]. Composites containing lead titanate [6,7], barium and strontium titanates [8,9] have been well studied. This approach allows to control the dielectric permittivity and losses by changing the concentration of ferroelectric inclusions in the composite. A distinctive feature of the approach is the uniform distribution of FE inclusions over the structure volume.

Another approach to the formation of ferroelectric composite structures is the introduction of ferroelectric particles into the pore space of glass matrices [10,11]. This approach allows to adjust the size, shape, and relative location of ferroelectric inclusions by choosing the type of matrix. It also provides the possibility of obtaining structures with an artificially created spatial distribution of the dielectric permittivity. The advantage of this approach is the possibility of creating materials with new properties: structures with a purposefully formed dispersion characteristic; structures with a specified distribution of submillimeter-sized inhomogeneities, which determine their frequency and spatial selectivity when interacting with electromagnetic waves; structures with any specified permittivity with values from units to several hundred for the implementation of complex functional devices of microwave electronics, in which the matching of individual elements will be achieved due to a gradient change in the permittivity of the substrate.

A promising way to create glass-ceramic structures for microwave applications is the introduction of barium-strontium titanate Ba_x_Sr_1–x_TiO_3_ (BST) into the pore space of a glass oxide matrix [12]. By varying the component composition of the solid solution, the permittivity of the composite can be changed within a wide range [13]. Oxide porous glasses exhibit thermal and chemical resistance, stable permittivity and low losses [14,15], which distinguishes them from other porous materials.

To obtain porous glass materials the following methods are used: through chemical etching (leaching) of two-phase glass with interpenetrating phases [16], the sol-gel method [17], template synthesis method [18] and replica method [19], which consists in sequential mixing of glass powder, solution for impregnation of the polymer matrix with further high temperature treatment of the finished mixture. The disadvantages of these methods are rather strict limitations on the pore size.

An alternative to the above approaches is a method for producing silicate glasses with a porous structure due to ion exchange between alkaline glass cations and salt melt cations [20], which allows one to control the size of pores in a wide range. The advantage of this method is the possibility of obtaining new porous glass materials with the required distribution of properties by volume, depending on the composition of the glass and the salt melt, the ionic radii of the exchanging cations, and the temperature and time of interaction.

The aim of this paper is to study the possibilities of creating glass-ceramic ferroelectric structures based on barium-strontium titanate, introduced in the pore space of silicate glass, formed by ion exchange between alkaline glass cations and salt melt, and to characterize their structure and electrical properties at microwaves.

## 2. Materials and Methods

### 2.1. Materials Synthesis and Characterization

The synthesis of potassium-iron-silicate glass (KFeSi glass) of the composition 15K_2_O-20Fe_2_O_3_-65SiO_2_, mol.% was carried out from chemically pure reagents-K_2_CO_3_, FeO, Fe_2_O_3_ and SiO_2_ in a platinum crucible at a temperature of 1450 °C for 2 h, followed by annealing at a temperature of 550 °C. Glass samples were quenched in air by casting the melt on a steel mold.

In order to form porous glasses, ion exchange processing of model glass plates was performed in NaNO_3_ and LiNO_3_ melts at temperatures of 450 °C with isothermal exposure for 9 h. The duration of isothermal treatment was chosen in such a way as to provide thorough processing of the glass plate, taking into account the data obtained earlier [21]. Since K^+^ ions are replaced by Li^+^ and Na^+^ due to ion exchange, the glass processed in LiNO_3_ and in NaNO_3_ will be designated as Li_2_O-Fe_2_O_3_-SiO_2_ (LiFeSi) and as Na_2_O-Fe_2_O_3_-SiO_2_ (NaFeSi), respectively.

The geometric parameters of the porous structure of glass after ion exchange and subsequent removal of the salt melt by treatment in water were determined by reference porometry of glass samples in the form of a disk with a diameter of 23 mm and a thickness of 2 mm, at the Porotech 3.1 installation in the resource center of St. Petersburg state University “Thermogravimetric and calorimetric research methods”. Electronic micrographs of the studied glass samples were obtained using a Zeiss SUPRA 40VP scanning electron microscope (Carl Zeiss, Munich, Germany) with an SE2 detector (SEM) in the resource center of St. Petersburg state University “Nanotechnologies”.

X-ray diffraction images (XRD) of samples were obtained using a DRON-3 diffractometer (radiation wavelength 0.154 nm, Cu K_α_) (Burevestnik, St. Petersburg, Russia) at room temperature.

The degree of iron oxidation in the glasses was determined by Mössbauer spectroscopy (nuclear gamma resonance) on the MC-1107 Em spectrometer (Institute for Analytical Instrumentation RAS, St. Petersburg, Russia). The samples were measured in the transmission mode with constant acceleration, using ^57^Co in the rhodium matrix as the source. The registered spectra were processed using a specialized MossFit software package.

In order to synthesis barium-strontium titanate (BST), the initial mixtures were prepared from hydrated titanium dioxide, which was obtained by the interaction between TiCl_4_ and dilute ammonia NH_4_OH at a reaction medium pH equal to 9.5. The residue was washed of impurities, and then dissolved in 1.4 mol/L solution of nitric acid. The solution of TiO(NO_3_)_2_ was obtained. The gravimetric method was used to control the concentration of TiO_2_ in the obtained solution of titanyl nitrate. It equaled 0.1 g/mL. Aqueous solutions of Ba(NO_3_)_2_ or Ba(CH_3_COO)_2_ and Sr(NO_3_)_2_ were introduced into the obtained solution of titanyl nitrate according to the stoichiometry of the forming complex oxide. Glycine (Vekton, St. Petersburg, Russia) was also added. The solutions containing amount of strontium nitrate were obtained corresponding to the calculated stoichiometry of Ba_0.7_Sr_0.3_TiO_3_. After the evaporation of excess water, porous glasses were placed in the sol and were kept in it at 80 °C within 2 h. After impregnation, the glass was heat treated at 500 °C within 3 h in air during which crystallization of barium-strontium titanate occurred with a low content of impurity phases both on the glass surface and in its pore space. The procedure of soaking a glass sample in the sol, followed by heat treatment, was repeated 2 times. After preparing glass samples with BST in a porous space, some of them were annealed in an oxygen stream at 500 °C for 2 h in order to increase the crystallinity of barium-strontium titanate.

### 2.2. Electrical Characterization

The dielectric (ε) permittivity of samples, as well as dependencies ε on frequency in microwaves were estimated by the Nicholson–Ross method [22]. For this purpose, a microstrip transmission line connected to the vector network analyzer was used. In this study the frequency dependences of the modulus of the reflection coefficient and the phase of the transmission coefficient were measured in the frequency range of 3–10 GHz, when samples were superimposed on the transmission line. Then, these measurement data were recalculated into dielectric permittivity. Dielectric losses (tan δ) of ceramic-glass structures were measured in plane-parallel samples with silver paste electrodes at a frequency of 1 MHz and room temperature by Agilent E4980A LCR meter (Agilent, Santa Clara, CA, USA).

## 3. Results and Discussion

### 3.1. Structural and Morphologic Characterization of Porous Glasses

**Figure 1:**
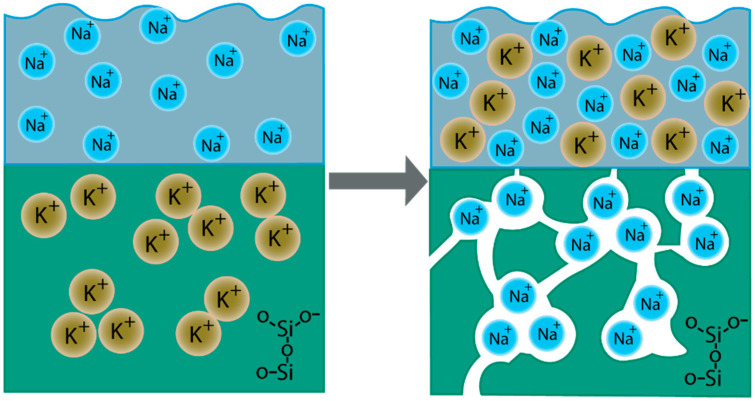
Ion exchange process between KFeSi glass and NaNO_3_ melt.

Ion exchange treatment of 15K_2_O-20Fe_2_O_3_-65SiO_2_ glass in a LiNO_3_ and NaNO_3_ melt at a temperature below the glass transition temperature (*T*_g_) leads to the formation of a porous structure in glass [21], as schematically shown in Figure 1 using the example of a KFS and NaNO_3_ interaction. In another study [23], it was established that the kinetics and nature of the interaction of glasses with molten salts substantially depend on the direction of the fluxes of exchanging cations. With the same ion exchange treatment, in the case where the flow of a cation with a smaller ionic radius is directed from the molten salt to glass, the interaction process leads to the formation of an opaque diffusion zone, the length of which is many times larger than the size of the zone during ion exchange with the opposite direction of alkaline cation fluxes. The reason for the observed anomalies of the ion exchange process is that at temperatures below the glass transition temperature silicon-oxygen groups cannot change their orientation and position in space. Replacing the alkaline glass cations with cations with a smaller ionic radius and, accordingly, with a higher field strength leads to tensile stresses at *T* < *T*_g_.

**Figure 2:**
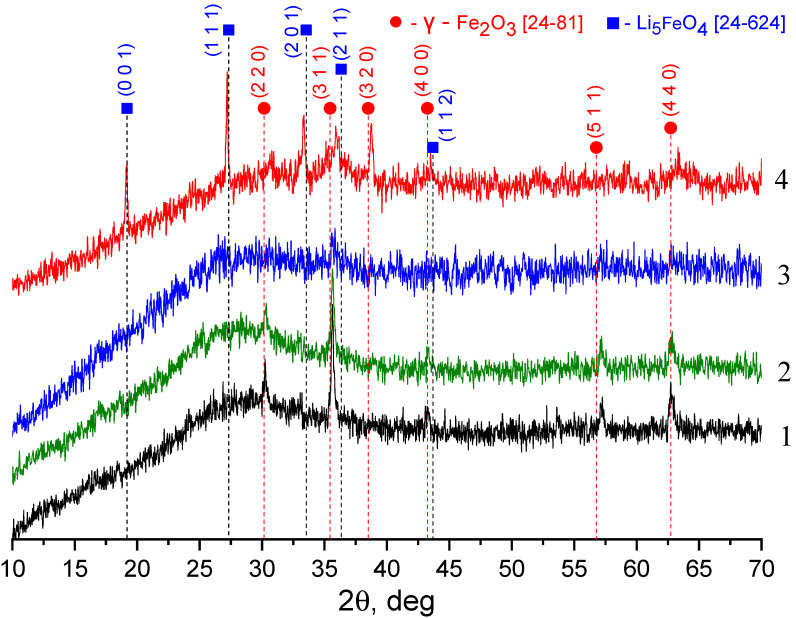
X-ray diffraction images (XRD) patterns of KFS glass samples: 1—tempered glass; 2—annealed glass; 3—glass after the ion exchange treatment in NaNO_3_ melt at 450 °C for 9 h; 4—glass after the ion exchange treatment in LiNO_3_ melt at 450 °C for 9 h (●—γ-Fe_2_O_3_ Pdf No 24-81 [24], ■—Li_5_FeO_4_ Pdf No 24-624 [24]).

Figure 2 shows diffractograms of annealed and tempered KFeSi glass, as well as glasses subjected to ion exchange treatment in LiNO_3_ and NaNO_3_ melts. XRD analysis data indicate that ion exchange in NaNO_3_ and LiNO_3_ melts has different effects on the formation of crystal phases in the samples under study. Figure 2 indicates the presence of crystalline iron oxide, presumably γ-Fe_2_O_3_, both in the initial glass (curves 1 and 2) and in the porous glass after ion exchange (curves three and four). The ion exchange procedure, which takes place at a temperature of 450 °C for 9 h, leads to a change in the phase composition and crystal structure of iron oxide: for both porous glasses, the intensity of reflexes decreases for most phases, for LiFeSi glass a rather intense peak (320) appears. Comparison of curves two and three allows us to suggest that ion exchange in the NaNO_3_ melt leads to a decrease in the content of the crystalline phase of iron oxide in the NaFeSi glass. On the contrary, the presence of rather intense reflexes (311), (320), and (440) in LiFeSi porous glass indicates a redistribution of the crystalline phases of iron oxide as a result of ion exchange (comparison of curves two and four). In addition to γ-Fe_2_O_3_, the X-ray image of LiFeSi glass shows reflexes indicating the presence of the Li_5_FeO_4_ phase.

It is known that magnetite (Fe_3_O_4_) and maghemite (γ-Fe_2_O_3_) cannot be distinguished by the X-ray method, because both oxides have a spinel-type structure. To identify the phases of iron oxides in the studied glasses, the Mössbauer spectroscopy method was used, since the parameters of the hyperfine interaction of the Mössbauer spectra of magnetite and maghemite differ significantly.

**Figure 3:**
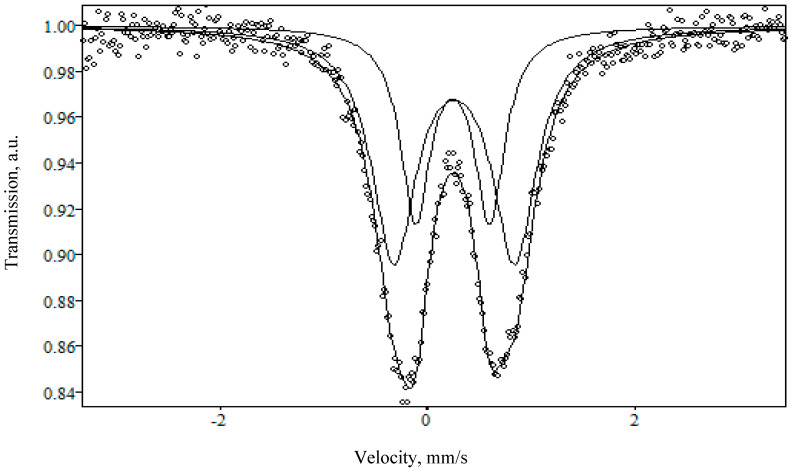
Mössbauer spectrum of the NaFeSi sample obtained at room temperature and in an external magnetic field.

**Figure 4:**
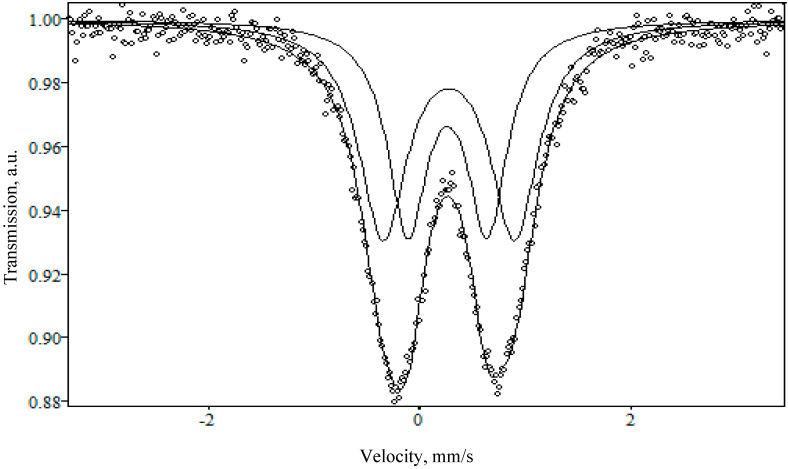
Mössbauer spectrum of the LiFeSi sample obtained at room temperature and in an external magnetic field.

Figure 3 and Figure 4 show the Mössbauer spectra of NaFeSi and LiFeSi glasses, respectively. Table 1 below shows hyperfine parameters: isomeric (or chemical) shift (IS), quadrupole splitting (QS), and line widths at half height (G). It can be seen from the presented data, that the spectra of the studied glasses are a superposition of two quadrupole doublets. The values of the isomeric shift suggest that all iron atoms are in the +3 valence state in unsymmetrical surroundings in both NaFeSi and LiFeSi glasses.

As shown in another study [25], the Fe^3+^ cations appear to be in tetrahedral surroundings. Sufficiently large values of quadrupole splitting indicate a significant distortion of polyhedra in the glass structure. It follows that iron Fe^+3^ is present in the studied glasses both as part of the crystalline oxide γ-Fe_2_O_3_ and directly in the glass structure.

The difference between the spectra lies in the shape of the doublets: in the case of the NaFeSi sample, the doublet has a more symmetrical shape than for LiFeSi, which indicates a more crystalline structure of the latter.

**Figure 5:**
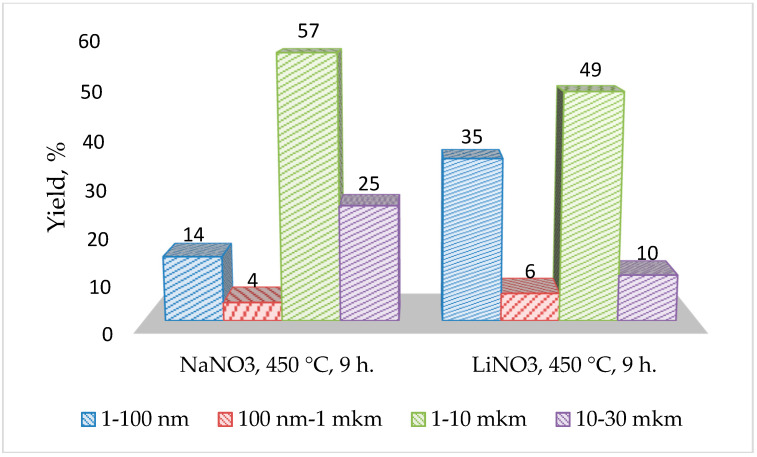
The distribution of the pore sizes for glasses after the ion exchange in NaNO_3_ and LiNO_3_ melts.

Figure 5 shows data on pore sizes in NaFeSi and LiFeSi glasses obtained by reference porometry. Analysis of porometric data shows that at an ion exchange temperature of 450 °C, 14% of the pores in the NaFeSi glass have a size in the range of 1–100 nm, and 57% in the range of 1–10 microns; in LiFeSi glass, the content of pores with a size in the range of 1–10 nm is 35%, and in the range of 1–10 microns—49%. The porosity values of these glasses by weight are 0.033 cm^3^/g for NaFeSi and 0.041 cm^3^/g for LiFeSi, with a specific surface area of 3.07 m^2^/g and 5.0 m^2^/g, respectively.

### 3.2. Characterization of BST in Porous Space of Glass

**Figure 6:**
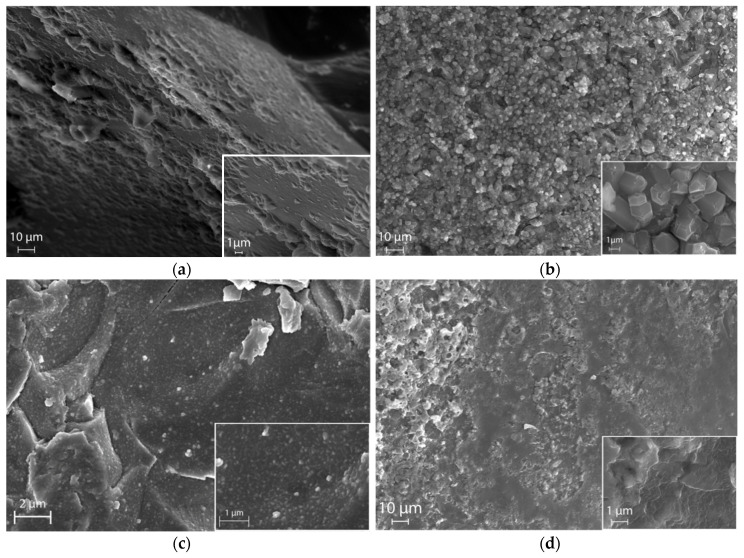
SEM-image of the surface of the original KFeSi (**a**); LiFeSi glass obtained as a result of ion exchange in LiNO_3_ (**b**); NaFeSi glass obtained as a result of ion exchange in NaNO_3_ (**c**); NaFeSi-based composite containing BaSrTiO_3_ (**d**).

Micrographs of the surface of the original KFeSi glass (a), LiFeSi (b) and NaFeSi (c) glasses obtained as a result of ion exchange, as well as NaFeSi glass (d) with embedded barium-strontium titanate are shown in Figure 6. According to SEM analysis data, the morphology of the glass surface after ion exchange treatment is in good agreement with the data of standard reference porometry. The micrographs allow us to estimate changes in the surface morphology of the original glass as a result of ion exchange in NaNO_3_ and LiNO_3_ melts and as a result of the introduction of barium-strontium titanate into the pore space of the glass matrix. SEM data indicate that the ion exchange of potassium with lithium and with natrium changes the surface of the original glass in different ways (see Figure 6b,c). Crystal structures that can be attributed to the orthorhombic phase of Li_5_FeO_4_ are clearly visible on the surface of LiFeSi glass. The micrograph of NaFeSi glass shows the presence of a fine-grained structure formed as a result of ion exchange. Based on the porometric data and the results of electron microscopy, it can be assumed that the glass subjected to ion exchange in the LiNO_3_ melt shows a more developed porous structure, which can have a positive effect on the efficiency of introducing barium-strontium titanate into it. Figure 6d indicates that the impregnation of porous glasses with BST sol leads to uniform pore filling and the formation of a surface without significant cavities.

**Figure 7:**
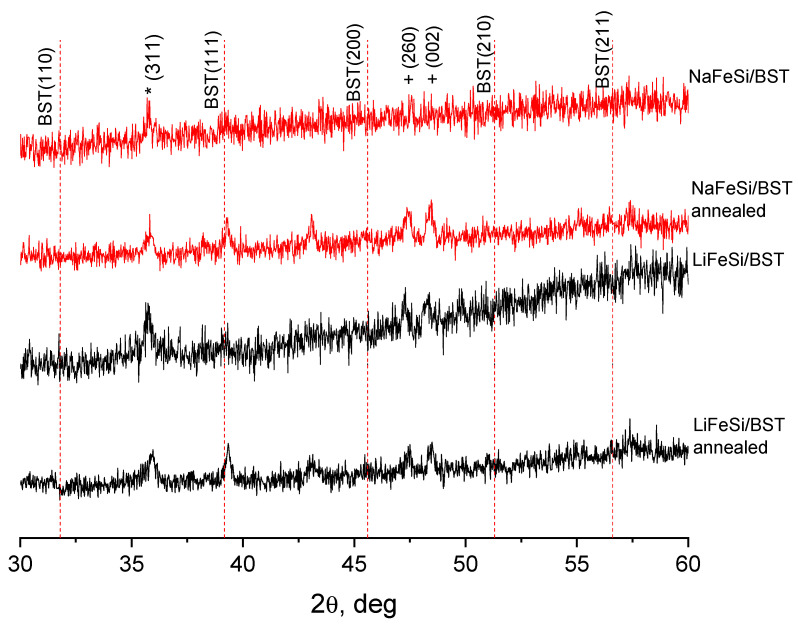
XRD of BaSrTiO_3_ in porous glass matrices before and after annealing in oxygen atmosphere (∗—γ-Fe_2_O_3_ Pdf No 24-81 [24]; +—presumably BaTi_4_O_9_ Pdf No 34-70 [24]).

Figure 7 shows diffractograms of NaFeSi/BST and LiFeSi/BST glass-ceramic samples before and after annealing in an oxygen atmosphere. Since the matrix glass is an X-ray amorphous material, the low-intensity peaks from barium-strontium titanate in the X-ray diffraction pattern are due to the fact that the pore volume is not completely filled with the crystalline phase, which is a typical situation for porous structures. From the XRD analysis data, it follows that high-temperature treatment in an oxygen atmosphere has a positive effect on the crystal structure of barium-strontium titanate—the diffractograms of samples after annealing clearly show the increase in intensity of (111) reflexes. This allows us to expect an improvement in the dielectric characteristics of glass-ceramic structures as a result of annealing—the increase in the dielectric constant and reduction in losses. In addition to BST peaks, the XRD images of glasses both before and after annealing show reflexes indicating the presence of the γ-Fe_2_O_3_ phase (marked as ∗) with an almost unchanged intensity.

Attention is drawn to the appearance of the more pronounced and intensive reflexes at the angles of 47° and 48° on the diffractograms of samples subjected to high-temperature processing (indicated as +) which may be due to the presence of crystal phases of secondary barium polytitanate in the composite, presumably BaTi_4_O_9_. The increase in polytitanate reflex intensity can be explained by the partial conversion of barium titanate to polytitanates in an oxygen medium [26]. Barium polytitanates, such as BaTi_2_O_5_, BaTi_3_O_7_, BaTi_4_O_9_ and so on, are intermediate products that can be formed in a stoichiometric mixture of reagents, depending on the temperature conditions of the synthesis process. Many of polytitanates have a crystal structure similar to BaTiO_3_, which makes it difficult to identify them by XRD. Generally being linear dielectrics with a permittivity scale of 10–40, these compounds have a greater influence on the final electrical properties of the composite, the greater their content in it [27].

### 3.3. Electrical Characterization of BST-Glass Structures

**Figure 8:**
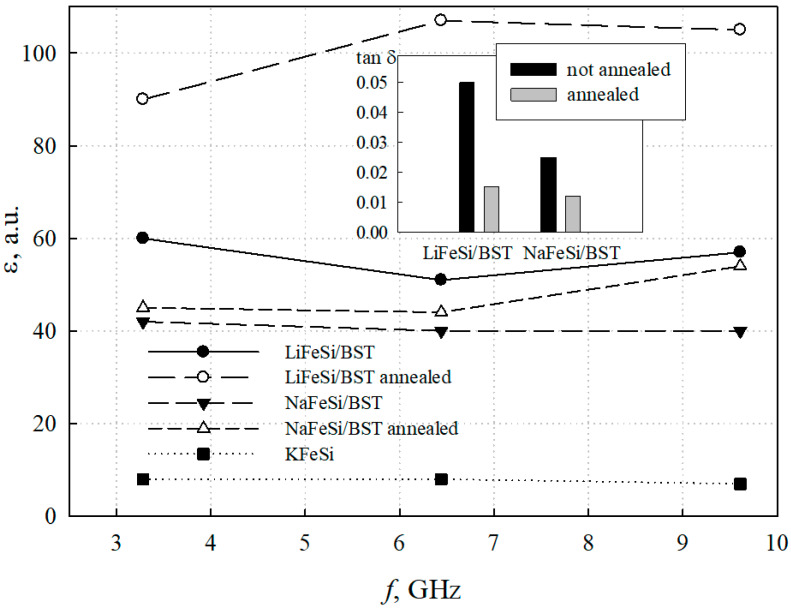
Dielectric permittivity of KFeSi base glass, and NaFeSi/BST and LiFeSi/BST glass-ceramic samples. The insert presents dielectric losses of NaFeSi/BST and LiFeSi/BST glass-ceramic samples before and after annealing in an oxygen atmosphere.

The frequency dependences of the permittivity of the base KFeSi glass, as well as NaFeSi/BST and LiFeSi/BST glass-ceramic samples before and after annealing in an oxygen environment are shown in Figure 8. It can be seen from the presented data that in the studied frequency range, the permittivity of both NaFeSi/BST and LiFeSi/BST samples weakly depends on the frequency. The NaFeSi/BST structure shows a permittivity of about 40, which increases slightly after annealing. The LiFeSi/BST one exhibits a significantly higher permittivity and shows an almost two-fold increase in it as a result of annealing. The increase in the permittivity is obviously related to the improvement in the crystal structure of the barium-strontium titanate as a result of annealing in an oxygen atmosphere [28,29]. The decrease in dielectric permittivity under the applied DC voltage was about 1% under electric field strength of 10 V/cm.

The decrease in tan δ of the glass-ceramic samples after their high-temperature treatment in O_2_ is shown in the insert of Figure 8. One of the possible explanations for this improvement in dielectric losses both for NaFeSi/BST and for LiFeSi/BST structures may be due to a decrease in the number of oxygen vacancies in the perovskite lattice of BST. It is known that barium-strontium titanate is often deficient in oxygen, i.e., there are vacancies in the oxygen sublattice [30]. When located near oxygen vacancies, titanium ions Ti^4+^ can capture free electrons and turn into Ti^3+^. The presence of Ti^3+^ ions in the lattice leads to the appearance of “hopping” conductivity, when electrons can move between different Ti^3+^ titanium ions and increase dielectric losses. It is obvious that annealing in an oxygen atmosphere restores the missing oxygen in the crystal lattice and, as a result, reduces losses by two to three times. A change in the charge state of iron ions (as a result of oxidation from Fe^3+^ to Fe^4+^) may be another possible reason for the change in losses due to high-temperature treatment of iron-containing samples in oxygen [31,32,33].

### 3.4. Porous Matrix Filling Estimation

Under various technological regimes of forming a composite system, the ferroelectric filler can impregnate a part of the porous matrix, leaving some inner pore layers free. In this case, the dielectric constant of the composite structure turns out to be lower than expected. In order to estimate the average penetration depth (*h*) of a ferroelectric filler into a porous matrix with the *H* thickness, the following analytical models can be used.

**Figure 9:**
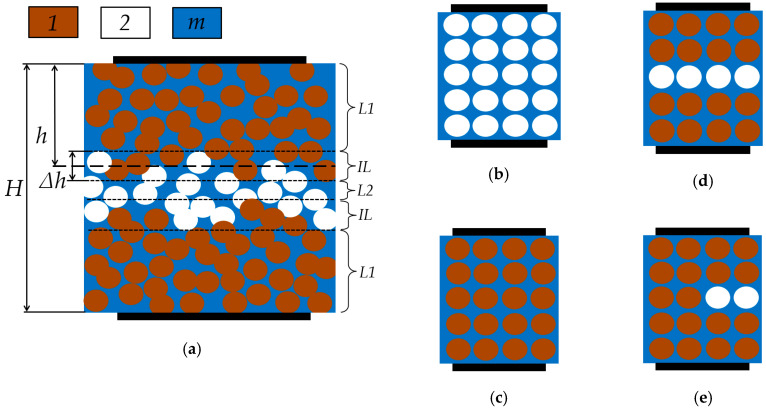
The composite film model (**a**) and its simplified particular cases: only porous matrix (**b**); all pores are filled (**c**); a model where the intermediate layer can be neglected (**d**); a model where the central layer is particularly filled (**e**), where *1* is ferroelectric, *2* is air, *m* is medium matrix.

Figure 9a schematically shows a typical case. The composite sample in general can be conditionally divided into several layers. Near-surface layers (*L1*) are a ferroelectric-filled glass matrix, since the pore filling process occurs on both sides. The central layer (*L2*) of the porous structure can be left empty if the technological process of pore filling is not optimal, and between these layers, there are intermediate layers (*IL*), in which some parts of the pores are occupied by the ferroelectric phase, and the other parts of the pores remains empty. This multilayer model is the most common case for the technology used in this study. Several simpler situations are shown in Figure 9b–e: only porous matrix (Figure 9b); all pores are filled with ferroelectric (Figure 9c); a model, when the intermediate layer can be neglected (Figure 9d); a model, where the central layer is particularly filled (Figure 9e). Electrodes are located above and below the composite film, forming a capacitor.

For the two-phase air/glass (Figure 9b) and ferroelectric/glass (Figure 9c) systems, the effective dielectric constant (ε_jm_) can be calculated in different ways if the volume fraction of the dispersed phase (φ) and the dielectric constants of the dispersed phase (ε_j_) and medium matrix (ε_m_) are known. Various models are used for estimating the effective dielectric constant. Different models have to be used to correctly describe the effects under consideration, since the effective dielectric properties of the composite depend on the peculiarities of phase mixing and the nature of their interaction, the filler concentration, its morphology, and the microstructure of the composite. Many of the above aspects depend on the technological conditions for obtaining the composite [34,35,36,37].

The simplest mixing model gives [37]:

(1) $$\varepsilon_{\mathrm{jm}}^{n}\left(\omega \right)=\varepsilon_{{}_{m}}^{n}\left(\omega \right)\left(\varphi -1\right)+\varepsilon_{{}_{j}}^{n}\left(\omega \right)\varphi ,$$

where ω is the frequency; *n* is the empirical parameter and ranges from −1 to 1, depending on composite morphology. For *n* equal to one, this is the model of an ideal mechanical mixture [38]. The deviation of *n* from unity corresponds to the deviation of the mixture from the ideal case. In practice, *n* is determined from the experiment, by approximating the experimental data by Equation (1), for this reason *n* is called the empirical parameter. For the model in Figure 9, the index j is one or two for ferroelectric- or air-filled (i.e., pores) glass, respectively.

Another model of the dielectric constant of a two-phase system is represented by the equation [38]:

(2) $$\varepsilon_{\mathrm{jm}}=\varepsilon_{j}\left(\omega \right)\left[1+\frac{k\varphi \left(\varepsilon_{m}\left(\omega \right)-\varepsilon_{j}\left(\omega \right)\right)}{\left(1-\varphi \right)\left(\varepsilon_{m}\left(\omega \right)-\varepsilon_{j}\left(\omega \right)\right)+k\varepsilon_{m}\left(\omega \right)}\right],$$

where *k* can vary greatly and relates to the filler microstructure [39,40,41]. When *k* is equal to three, it gives a Maxwell–Garnett equation [42], which is a model based on the analysis of the average field values for a single spherical inclusion in a continuous medium of a polymer matrix. In another study [31], it was established that the composite sample was satisfactorily described with *k* equal to 20.

The Bruggeman model gives the following equation [43]:

(3) $$\left(1-\varphi \right)\frac{\varepsilon_{m}\left(\omega \right)-\varepsilon_{\mathrm{jm}}\left(\omega \right)}{\varepsilon_{m}\left(\omega \right)+2\varepsilon_{\mathrm{jm}}\left(\omega \right)}+\varphi \frac{\varepsilon_{j}\left(\omega \right)-\varepsilon_{\mathrm{jm}}\left(\omega \right)}{\varepsilon_{j}\left(\omega \right)+2\varepsilon_{\mathrm{jm}}\left(\omega \right)}=0.$$

This model is based on the mean field theory and considers the composite in the form of repeating elements consisting of a matrix phase containing the inclusion of a spherical filler in the center. 

The effective dielectric constant ε_eff_ of the complete three-phase system of the multilayer model (Figure 9a) is determined by the dielectric properties of each of the layers and their thicknesses, according to Equation (4):

(4) $$\frac{H}{\varepsilon_{\mathrm{eff}}\left(\omega \right)}=\frac{2h-\Delta h}{\varepsilon_{1m}\left(\omega \right)}+\frac{4\Delta h}{\varepsilon_{1m}\left(\omega \right)+\varepsilon_{2m}\left(\omega \right)}+\frac{H-2h-\Delta h}{\varepsilon_{2m}\left(\omega \right)},$$

where indices one, two and *m* refer to the ferroelectric phase, air and matrix medium, respectively; ε_1m_ and ε_2m_ are the effective dielectric constant of the ferroelectric/matrix and porous matrix, respectively; ω is the circular frequency; *H*, *h*, Δ*h* are the thickness of the composite film, the average penetration depth of ferroelectric filler and the thickness of the intermediate layers, respectively. Equation (4) was derived from geometric considerations based on the sequence of layer arrangements shown in Figure 9a.

For this model the values of ε_1m_ and ε_2m_ can be calculated according to one of the Equations (1)–(3), for which the volume fraction of the dispersed phase will be the same and equal to the volume fraction of pores in the glass matrix.

The thickness of the intermediate layer can be estimated from considerations of the average statistical fluctuation theory [44] and diffusion limitation of the process [45] of introducing a ferroelectric filler into a porous matrix, according to Equation (5):

(5) $$\Delta h=h/\sqrt{N},\;N\approx \varphi N_{1}\sqrt{D_{1}\tau }/H,$$

where *N* is the number of filler microstructural particles involved in the process; *N*_1_ and *D*_1_ are the total number and diffusion coefficient of the filler particles; *τ* is the processing time.

Solving the system of the equations above, it is possible to estimate the process efficiency of the composite formation, determining how deeply the ferroelectric filler was introduced into the porous matrix.

Equation (4) can be simplified by replacing the three-phase system of the general multilayer model (Figure 9a) with the special ones shown in Figure 9d,e:

(6) $$\frac{H}{\varepsilon_{\mathrm{eff}}\left(\omega \right)}=\frac{2h}{\varepsilon_{1m}\left(\omega \right)}+\frac{H-2h}{\varepsilon_{2m}\left(\omega \right)};\;\frac{H}{\varepsilon_{\mathrm{eff}}\left(\omega \right)}=\frac{4h-H}{\varepsilon_{1m}\left(\omega \right)}+\frac{4\left(H-2h\right)}{\varepsilon_{1m}\left(\omega \right)+\varepsilon_{2m}\left(\omega \right)},$$

where the left equation corresponds to the case of a series layer model of completely filled and empty pores, and the right equation corresponds to the case of a half-filled central layer.

By solving Equation (4) or (6), as well as substituting one of Equations (1)–(3) in them, depending on the choice of the model, it is possible to estimate the penetration depth (*h*).

For the series layer model (Figure 9d) the penetration depth using Equation (1) can be estimated as:

(7) $$h=\frac{H}{2}\cdot \frac{\left(\varepsilon_{m}^{n}\left(\omega \right)\left(\varphi -1\right)+\varepsilon_{1}^{n}\left(\omega \right)\varphi \right)\left(\varepsilon_{{}_{m}}^{n}\left(\omega \right)\left(\varphi -1\right)+\varepsilon_{2}^{n}\left(\omega \right)\varphi -\varepsilon_{\mathrm{eff}}\left(\omega \right)\right)}{\varepsilon_{\mathrm{eff}}\left(\omega \right)\varphi \left(\varepsilon_{2}^{n}\left(\omega \right)-\varepsilon_{1}^{n}\left(\omega \right)\right)}.$$

For the series/parallel layer model (Figure 9e), in which the near-surface layers are completely filled with ferroelectric and the central one is half filled, the penetration depth can be estimated as:

(8) $$h=\frac{H}{2}\cdot \frac{\left(\varepsilon_{{}_{m}}^{n}\left(\varphi -1\right)+\varepsilon_{1}^{n}\varphi \right)\left(2\varepsilon_{{}_{m}}^{n}\left(\varphi -1\right)+\left(\varepsilon_{1}^{n}+\varepsilon_{2}^{n}\right)\varphi \right)+\varepsilon_{\mathrm{eff}}\left(2\varepsilon_{{}_{m}}^{n}\left(\varphi +1\right)+\left(\varepsilon_{2}^{n}-3\varepsilon_{1}^{n}\right)\varphi \right)}{2\varepsilon_{\mathrm{eff}}\varphi \left(\varepsilon_{2}^{n}-\varepsilon_{1}^{n}\right)}.$$

On the basis of the considered Equations (1)–(8), the degree of filling of the porous glass matrix by BST were estimated, depending on the type of matrix and the treatment modes of the sample. The obtained parameters are shown in Table 2. The spread in the values of 2*h*/*H* is determined by the different models used.

The results show the importance of annealing, which apparently improves the degree of impregnation of the porous system with the ferroelectric filler.

## 4. Conclusions

Glass-ceramic structures based on porous NaFeSi and LiFeSi glasses and barium-strontium titanate incorporated in the porous space were formed and studied using XRD, SEM, and porometry methods. The identification of iron oxide in the glasses investigated was facilitated by Mössbauer spectroscopy. Electrical characteristics such as permittivity and losses of as-prepared and annealed in oxygen medium samples were also investigated in microwaves.

The results of the studies conducted allow us to draw the following conclusions.

1. The method of obtaining porous glasses due to ion exchange between KFS glass and LiNO_3_ and NaNO_3_ melts allowed us to control a wide range of pore sizes and made it possible to form glass porous structures with pores of the required size. The ion exchange treatment between the cations of potassium-containing glass and NaNO_3_ and LiNO_3_ melts leads to the formation of porous structures with significantly different pore size distributions, which affects the efficiency of the barium-strontium titanate insertion in a porous space and, consequently, the electrical characteristics of the structures.
2. XRD data indicate the presence of crystalline iron oxide, both in the initial glass and in the porous glass after ion exchange. The ion exchange procedure leads to a change in the phase composition and crystal structure of iron oxide. The ion exchange in the NaNO_3_ melt leads to a decrease in the content of the crystalline phase of iron oxide in the NaFeSi glass. On the contrary, the presence of rather intense reflexes of iron oxide in LiFeSi porous glass indicates a redistribution of the crystalline phases of iron oxide as a result of ion exchange. The charge state of iron in porous glass, and, consequently, the type of iron oxide—γ-Fe_2_O_3_ was determinated by Mössbauer spectroscopy.
3. Based on the porometric data and the results of electron microscopy, it can be assumed that the glass subjected to ion exchange in the LiNO_3_ melt shows a more developed porous structure, which has a positive effect on the efficiency of introducing barium-strontium titanate into it. The impregnation of porous glasses with BST sol leads to uniform pore filling and the formation of a rather uniform surface without significant cavities.
4. Based on porous silicate glasses obtained by ion exchange, glass-ceramic materials containing a solid solution of barium-strontium titanate with a dielectric constant of more than 100 at microwaves were synthesized for the first time. The resulting glass-ceramic structures were characterized by dielectric permittivity and losses, depending on the method of obtaining porous glass and high-temperature processing in an oxygen environment. An increase in the intensity of BST peak (111), an increase in permittivity, and a decrease in losses of glass-ceramic samples after annealing in oxygen confirm the presence of crystalline BST in the pore space of NaFeSi and LiFeSi glasses.
5. Annealing of glass-ceramic structures in an oxygen environment has a positive effect on their electrical characteristics. NaFeSi/BST glass-ceramic structures demonstrate a slight increase in the permittivity and a decrease in losses from 0.025 until 0.01 after the high-temperature treatment in oxygen. LiFeSi/BST structures look more preferable: the permittivity increases from 60 to 100, while the losses reduce by more than three times, from 0.05 to 0.02 as a result of annealing.

## Figures and Tables

**Table 1:** Hyperfine parameters obtained from Mössbauer spectrum of the NaFeSi and LiFeSi samples.

Sample	#	G (mm/s)	IS (mm/s)	QS (mm/s)	%
NaFeSi	1	0.514 ± 0.025	0.249 ± 0.004	1.163 ± 0.040	64.00
	2	0.351 ± 0.031	0.232 ± 0.004	0.714 ± 0.024	36.00
LiFeSi	1	0.550 ± 0.024	0.266 ± 0.003	1.242 ± 0.036	55.88
	2	0.453 ± 0.026	0.254 ± 0.003	0.753 ± 0.027	44.12

**Table 2:** Parameters of the NaFeSi/BST and LiFeSi/BST samples.

Sample	Annealing	Concentration of Pores, cm^3^/g	ε_eff_ (9.7 GHz)	*2h/H*
LiFeSi/BST	No	0.041	58	0.84…0.94
	Yes	0.041	110	0.92…0.98
NaFeSi/BST	No	0.033	40	0.82…0.93
	Yes	0.033	55	0.91…0.97
